# Size, Stability, and Porosity of Mesoporous Nanoparticles Characterized with Light Scattering

**DOI:** 10.1186/s11671-017-1853-y

**Published:** 2017-01-25

**Authors:** Martti Kaasalainen, Vladimir Aseyev, Eva von Haartman, Didem Şen Karaman, Ermei Mäkilä, Heikki Tenhu, Jessica Rosenholm, Jarno Salonen

**Affiliations:** 1 Laboratory of Industrial Physics, Department of Physics and Astronomy, University of Turku, FI-20500 Turku, Finland; 2 Department of Chemistry, University of Helsinki, FI-00014 HY Helsinki, Finland; 3 Pharmaceutical Sciences Laboratory, Faculty of Science and Engineering, Åbo Akademi University, FI-20520 Turku, Finland

**Keywords:** Porosity, Porous silicon, Mesoporous silica nanoparticle, Light scattering, Particle size, Biorelevant characterization

## Abstract

Silicon-based mesoporous nanoparticles have been extensively studied to meet the challenges in the drug delivery. Functionality of these nanoparticles depends on their properties which are often changing as a function of particle size and surrounding medium. Widely used characterization methods, dynamic light scattering (DLS), and transmission electron microscope (TEM) have both their weaknesses. We hypothesize that conventional light scattering (LS) methods can be used for a rigorous characterization of medium sensitive nanoparticles’ properties, like size, stability, and porosity. Two fundamentally different silicon-based nanoparticles were made: porous silicon (PSi) from crystalline silicon and silica nanoparticles (SN) through sol-gel process. We studied the properties of these mesoporous nanoparticles with two different multiangle LS techniques, DLS and static light scattering (SLS), and compared the results to dry-state techniques, TEM, and nitrogen sorption. Comparison of particle radius from TEM and DLS revealed significant overestimation of the DLS result. Regarding to silica nanoparticles, the overestimation was attributed to agglomeration by analyzing radius of gyration and hydrodynamic radius. In case of PSi nanoparticles, strong correlation between LS result and specific surface area was found. Our results suggest that the multiangle LS methods could be used for the size, stability, and structure characterization of mesoporous nanoparticles.

**Electronic supplementary material:**

The online version of this article (doi:10.1186/s11671-017-1853-y) contains supplementary material, which is available to authorized users.

## Background

Nanoparticles’ properties are in key role when new biomedical applications are considered. Biodistribution of nanoparticles [1], their interactions with cell components [2], and protein corona formation [3] are determined by their properties. In addition, nanoparticles’ drug loading capacity, colloidal stability, and interactions with loaded drugs are related to their physico-chemical properties and are important for a functional drug delivery device [4–6]. On another point of view, these same properties are also affecting nanoparticles’ toxicity [7–9]. Few of the most important properties are size distribution, shape, charge, composition, purity, stability, and surface area [10]. Because particles are in nanoscale, properties can significantly differ from bulk properties which make it crucial to study these every time when physico-chemical modifications to nanoparticles are made [11]. The fact that nanoparticles are studied and used, in aqueous medium, binds these characteristics together; shape affects size distribution and charge affects stability, which again affects size distribution. In our previous studies, we have noticed that in many cases, the properties of nanoparticle formulation depend strongly on surrounding medium [12, 13]. The aqueous medium becomes more complex on biological systems where other compounds, such as cells and proteins, are present also. Studies with biorelevant characterization have been found to be inevitable in the development of biological nanotechnology [7, 10, 14, 15].

Dynamic light scattering (DLS) is widely used and preferred technique in characterization of nanoparticles on a simple solvent or biological environment [14, 16–18]. Non-invasive and fast measurement when sample is in its native colloidal state and good statistical significance of the result are the strengths of DLS [19]. These are particularly evident when compared to other common sizing technique, electron microscopy, which is usually expensive and time consuming, and in most cases, require the sample in a dry state. Nanoparticles’ drying increases the risk of changing the sample through shrinking, breaking down, or agglomeration and decreases the significance of the result, especially when biomedical applications are considered [20, 21]. On the other hand, electron microscopes can find details that strongly averaging DLS cannot. These could be, for example, particle morphology, porosity, and all particle populations in a polydisperse sample.

Widely used one angle DLS measurement does not solve the problem of determining the size of agglomerated sample. Very often, it is possible to measure an average particle size of the agglomerated colloid in question, but the primary particle size remains unresolved and the operator cannot even judge if the result is from agglomerates or primary particles. The poor resolution of DLS typically arises from the polydispersity of the sample, and this drawback can be reduced by using multiangular light scattering techniques. Multiangular light scattering is sensitive to polydispersity because the result is angle dependent if the particles are large enough [19]. Another indirect benefit of multiangular DLS is the ability to use another technique, static light scattering (SLS), at the same time. With SLS, information about mass-weighted particle size as well as particle shape and structure can be derived from the scattering intensity pattern.

The ability to gain information from the inner structure of particle is especially interesting in case of mesoporous nanoparticles. The high inner volume of a mesoporous matrix enables high payload of active molecules and the control over their release. The desired properties are achieved with particles’ physico-chemical modifications, and they depend on the type of the active molecule. In the case of oral peptide delivery, protection and sustained release of sensitive molecules are desired [22–24] and on the other hand, mesoporous particles can be used to stabilize the amorphous state of a drug [25–27]. In these applications, surface chemistry and pore dimensions need to be controlled [28, 29].

In this study, we compare the morphology, porosity, and size of silicon-based mesoporous nanoparticles by combining dry-state techniques like transmission electron microscope (TEM) and nitrogen sorption measurements to the multiangular light scattering (LS) techniques. The aim of this study is to find out if the drawbacks of DLS and TEM can be compensate or overcome with multiangular LS studies. We believe that the in future, LS techniques could help us and other researchers to characterize and study nanomaterials in more natural colloidal state and this way take us closer the characterization in biological, or at least more biorelevant, environment.

## Methods

### Preparation of Nanoparticles

Two different kinds of nanoparticles were used. Porous silicon (PSi) nanoparticles are top-down nanomaterial, fabricated by etching the porous film on the crystalline silicon wafer and by milling the film into nanoparticles. More detailed description of fabrication can be found from Additional file 1 and from references [1, 12, 30], for example. PSi nanoparticles with different specific surface areas were fabricated. The essential fabrication parameters are tabulated in Table 1. Surface area with type of the used silicon wafer is used on the naming of the PSi nanoparticles.

**Table 1:** Fabrication of the PSi nanoparticles

Sample	Substrate type	Current density	Total etch time	Illumination
		mA/cm^2^	s	
pPSi_190	p+	50/200	1200	–
nPSi_310	n+	30	1200	60 W, tungsten incandescent
nPSi_350	n+	75	1200	60 W, tungsten incandescent
nPSi_390	n+	75	1200	–
nPSi_480	n+	65	1200	100 W, tungsten halogen

Detailed description can be found from supplementary material

Bottom-up synthesis route was used with mesoporous silica nanoparticles (MSN) which were fabricated via controlled nucleation and growth of silica structure around self-assembled template of surfactants. Details of the syntheses can be found from Additional file 1 and in case of hollow MSN in [31]. Summary of fabrication details is shown in Additional file 1: Table S1.

With different PSi nanoparticles, the target was to vary pore volume and specific surface area while maintaining the pore morphology as constant as possible. Silica nanoparticles, in turn, were used in order to achieve more variations to pore morphology and orientation (Table 2). In the case of P-MSN, pores are aligned parallel to each other while with R-MSN pores are radially aligned, pointing towards the center of the particle. With H-MSN, core is hollow and radial porous structure is formed on the shell.

**Table 2:** Structure and pore morphology of the silica nanoparticles

Sample	Structure	Pore orientation
P-MSN	Mesoporous	Parallel to each other
R-MSN	Mesoporous	Radial
H-MSN	Mesoporous and hollow core	Radial
S-SN	Solid	–
L-SN	Solid	–

### Pore Volume and Specific Surface Area

Pore volume (V_p_) and specific surface area (SSA_BET_) were measured from the dried nanoparticle samples with nitrogen sorption measurements (TriStar 3000, Micromeritics Inc.). Specific surface area was calculated according to the BET theory [32], and pore volume was taken as the total adsorbed amount at a relative pressure *p*/*p*_0_ = 0.9 [33]. The nitrogen sorption measurements were made for all PSi nanoparticle samples but only for one silica nanoparticle sample since the porosity differences between silica samples were obvious already based on the different synthesis parameters. In addition, pore morphology was verified in electron microscope.

### TEM Analysis

Size and shape of nanoparticles were analyzed with TEM (JEM-1400 Plus, JEOL Ltd.) with 120 kV acceleration voltage. ImageJ 1.50 [34] was used for particle analysis with following procedure.

Originally, 8-bit grayscale image was turned to binary scale by adjusting the threshold with the “Threshold” tool. Threshold selection method was fixed inside one particle batch as the same thresholding method was impossible to use to all samples, due to the contrast differences arising from the size and density of nanoparticles. Particles were selected with “Analyze particles” tool. Smallest particles (area < 400nm^2^, *r* ≲ 12nm) were filtered out in order to exclude the false particle identifications from the image noise and defects in the supporting grid. In case of silica nanoparticles, circularity measure was used in order to exclude agglomerates from the analysis. The complicated morphology of the PSi nanoparticles made this method less convenient and, in this case, the agglomerated particles were identified visually.

In order to retain comparability of TEM to LS measurements, all samples except H-MSN were filtered with a 0.45-μm syringe filter (VWR International 25 mm with PTFE membrane).

Size data from the analysis was divided into 11 logarithmic bins, and Origin 8 software was used for log-normal fits. As a result, average size (*R*_*e*_) and geometric standard deviation (*σ*) are reported. Number of measured particles was sufficient (over 300) in all studied samples with the exception in nPSi_480 where 75 particles were measured because of the limited amount of sample (Additional file 1: Table S2).

### Static and Dynamic Light Scattering

Methodological aspects of static (SLS) and dynamic (DLS) light scattering can be found elsewhere [35]. LS measurements were made with a Brookhaven Instruments BI-200SM goniometer, a BIC-TurboCorr digital pseudo-cross-correlator, and a BI-CrossCorr detector, including two BIC-DS1 detectors. Either red or blue lasers were used depending on the nanoparticles absorbance. In case of silica nanoparticles, absorption of visible light is low, but in case of PSi nanoparticles, absorbance increases strongly when the shorter wavelengths are used (Additional file 1: Figure S1.). The unwanted effects of highly absorbing material were minimized by using a red 637 nm laser (A BIC Mini-L30 diode laser). In case of silica nanoparticles, a blue 488.0-nm laser (Coherent Sapphire laser 488-100 CDRH) was used. LS measurements were made from scattering angle 30° to 150° with 5° interval.

In DLS experiments, pseudo-cross-correlation functions of the scattered light intensity were collected using the self-beating scheme [35]. Correlation functions were analyzed with Cumulants algorithm, which gives a single average value of the translational diffusion coefficient (*D*_*t*_). Hydrodynamic radius (*R*_*h*_) is calculated from *D*_*t*_ of nanoparticles according to Stokes-Einstein equation

$$ {D}_t=\frac{k_B T}{6\pi \eta {R}_h}, $$

where *k*_*B*_ is Bolzmann’s constant, *T* is temperature, and *η* is medium viscosity. Temperature was set to 20 °C and controlled with a Lauda RC 6 CP thermostat. Viscosity of methanol was set to 0.591 cP and refractive index to 1.332.

The effective hydrodynamic radius (*R*_*h*_^eff^) was measured at a fixed scattering angle (*θ*) and a mass concentration of particles (*c*). The true hydrodynamic radius can then be obtained by extrapolating *R*_*h*_^eff^ to zero angle and zero concentration. Our experiments reveal negligible effect of particles concentration on *D*_*t*_ and thus on *R*_*h*_^eff^. Therefore, herein *R*_*h*_ refers to *R*_*h*_^eff^ extrapolated to zero angle.

SLS was used for determining the radius of gyration (*R*_*g*_) for the nanoparticles. *R*_*g*_ is a geometrical quantity, which is defined as a weight averaged root mean square distance of elements (and in this case, scattering centers) from the center of mass. Compared to hydrodynamic size, *R*_*g*_ is more sensitive to structure and geometry of the particle. By the definition, *R*_*g*_ for a sphere with radius *R* gives a relation, ${R}_g/ R=\sqrt{3/5}\approx 0.77$. If the particle is hollow, the *R*_*g*_/*R* approaches unity as the thickness of the shell approaches zero. If the particle is disc shaped ${R}_g/ R\hbox{'}=1/\sqrt{2}\approx 0.71$ when *R*’ is a radius of the disc [36].

Normalized scattering intensity from the particles *P*(*q*), i.e., the scattering function is defined as *P*(*q*) = *P*(*θ*) = *I*(*θ*)/*I*(*θ* = 0^°^), where *q* = (4*πη*_0_/*λ*) × sin (*θ*/2) is the scattering vector, *η*_0_ is the refractive index of the medium, *λ*_0_ is a wavelength in vacuum, and *θ* is the scattering angle. In other words, *P*(*q*) is calculated by subtracting the scattering of the medium from the total scattering intensity and normalizing this value to the intensity at the extrapolated angle *θ* = 0^°^. The Debye-Bueche [37, 38] or Guinier [39] scattering function was found to be the most suitable for *R*_*g*_ determination.

### Sample Preparation for LS Studies

Pure solvents used in LS experiment were filtered with a 0.2-μm syringe filter (Pall Acrodisc CR 13 mm with PTFE membrane). Cuvettes were first rinsed with methanol and then dried in filtered compressed air stream. In order to reduce the adsorption of positively charged silica particles onto the negatively charged glass surface, a cuvette was silanized with 5 vol-% mixture of APTES ((3-aminopropyl)triethoxysilane) and toluene. Background scattering of medium, methanol, was measured and subtracted. Samples were diluted to methanol as low concentration as possible in order to avoid agglomeration and multiple scattering. The sample was drawn into a syringe and filtered with a 0.45-μm syringe filter (VWR International 13 mm with PTFE membrane) in order to avoid dust particles. The exception to filtering step was made with the bigger hollow MSNs in which case the diluted suspension was used as prepared.

### Zeta Potential Measurements

Zeta potential measurements were made in order to find out the reason for agglomeration. Zeta potential was measured with electrophoretic light scattering using Malvern Zetasizer Nano ZS. Methanol was used as a medium, and zeta potential was calculated from the electrophoretic mobility with Hückel approximation, which is more suitable for nonaqueous solvents [40]. Measurement was repeated five times, and average values are reported.

## Results

### Particle Morphology

Different kinds of porous silicon (PSi) nanoparticles (Table 1) and silica nanoparticles (SN) (Additional file 1: Table S1 and Table 2) were fabricated in order to compare the effect of particle size, porosity, and morphology to light scattering (LS) results. Nitrogen adsorption measurements were carried out for all PSi nanoparticles and P-MSN particles, and results (Additional file 1: Figure S2) demonstrate a typical mesoporous adsorption behavior [41]. Porosity is typically defined as a ratio of pore volume to the total volume of the particle. The pore volume can be determined from nitrogen sorption measurements, but in our previous studies, we have noted that pore volume may be unreliable in the case of PSi nanoparticles. The pore volume can change significantly when nanoparticles are milled, as an example [42]. The specific surface area calculation is more straightforward and does not need an assumption of pore shape. Thus, it should be more repeatable and a better measure to compare mesoporous nanoparticles with different pore morphologies. Here, the specific surface area is used to quantify the morphology of the nanoparticles.

**Fig. 1:**
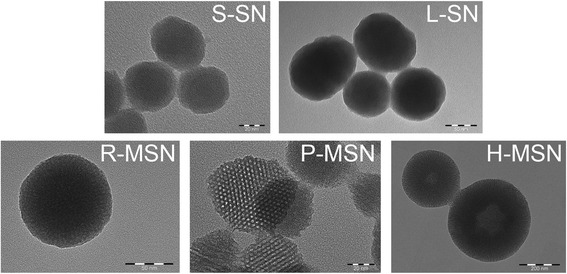
TEM pictures and descriptions of different silica nanoparticles used in the study

**Fig. 2:**
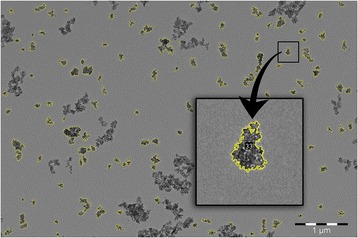
PSi nanoparticles with *yellow outline* are included in the distribution analysis from TEM pictures. *Inset* shows an example of the typical morphology of PSi nanoparticles

The bulky nature of nitrogen adsorption measurement (dry state and large required sample amount) is inconvenient when nanoparticles are considered. In the case of silica nanoparticles, the alignment of the pores was qualitatively characterized via TEM micrographs (Fig. 1 and Additional file 1: Figures S3–S5). For verification and comparison to PSi samples, nitrogen adsorption measurement was made for one SN sample also (Additional file 1: Figure S2). Silica and PSi nanoparticles showed a desired pore orientation and particle shape. Clear difference on particle shape between silica and PSi nanoparticles can be observed from Figs. 1 and 2.

### TEM Size Distributions

**Fig. 3:**
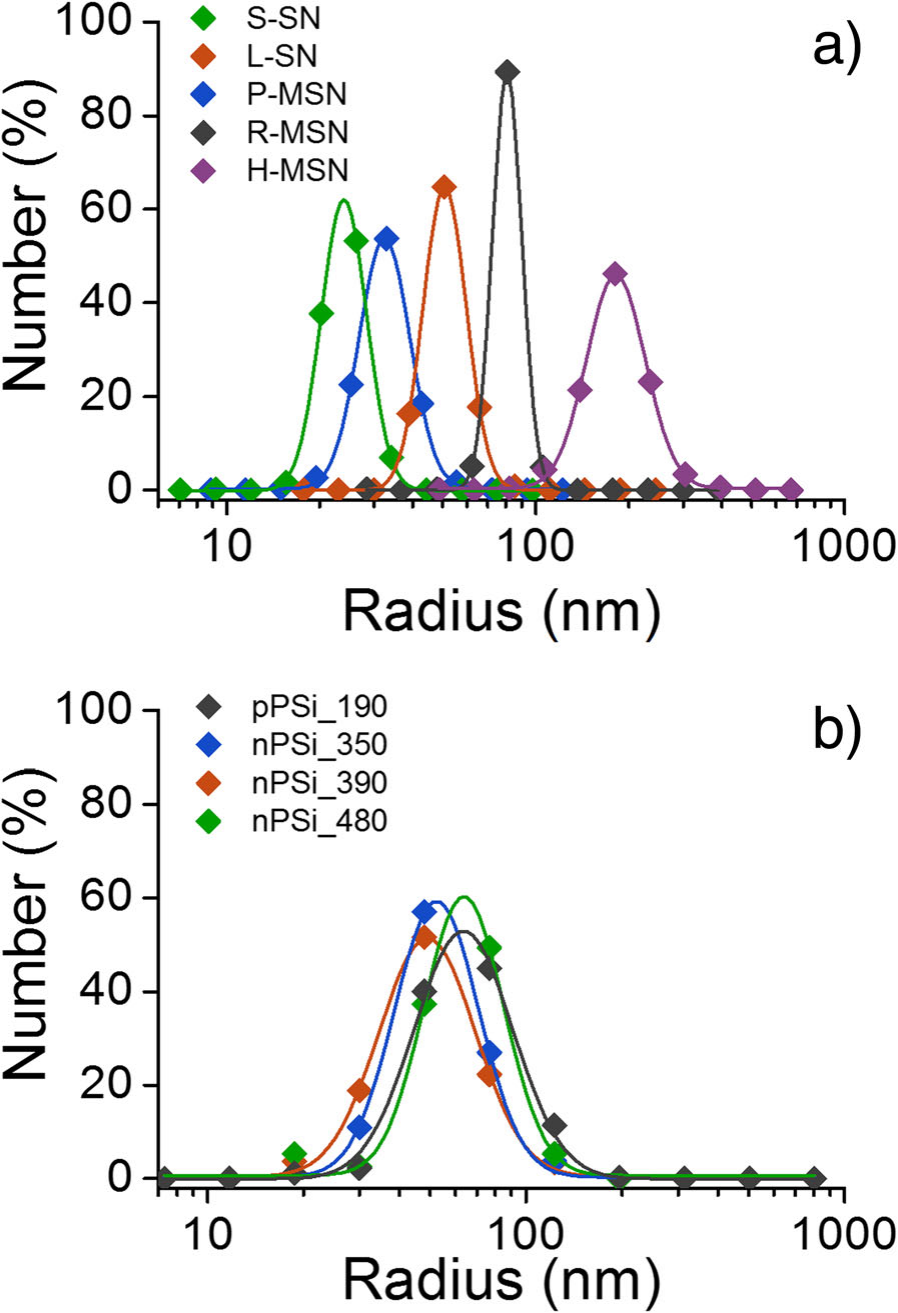
Particle size distribution of silica (**a**) and PSi (**b**) nanoparticles determined from TEM micrographs. Lines represent log-normal distribution fit to a corresponding particle batch

TEM images of PSi nanoparticles were analyzed from four different samples (Fig. 3). Obvious agglomerates were excluded from the analysis (Fig. 2). The morphology of PSi nanoparticles is irregular which leads to the fact that circularity cannot be used to distinguish primary particles. Particle size distribution was found to be similar in all studied particle batches, and the data fits reasonably well to log-normal distribution (Additional file 1: Table S2) as also observed before in [30].

In case of silica nanoparticles, the spherical shape and more homogenous primary particle size distribution enabled the statistical separation of primary particles from non-spherical agglomerates. The results are shown in Fig. 3 where the clear size difference between silica nanoparticles can be seen. Log-normal distributions fitted well to the data (Additional file 1: Table S2). Sample R-MSN shows the narrowest particle size distribution and sample H-MSN the widest.

The average primary particle sizes (*R*_*e*_) and geometric standard deviations (*σ*) derived from log-normal distribution fits are reported on Table 3.

**Table 3:** Average sizes, comparisons, and specific surface area of PSi and silica nanoparticles

Sample	*R*_e_	*σ*	*R*_g_	*R*_h_	*R*_h_/*R*_e_	*R*_g_/*R*_h_	SSA_BET_
	nm		nm	nm			m^2^/g
pPSi_190	72.1	0.36	80.2	94.1	1.31	0.85	188
nPSi_390	55.3	0.35	93.5	91.2	1.65	1.03	393
nPSi_350	57.5	0.30	81.8	84.4	1.47	0.97	354
nPSi_310	–	–	81.3	86.6	–	0.94	312
nPSi_480	69.3	0.29	98.8	97.1	1.40	1.02	475
P-MSN	33.6	0.19	78.6	75.9	2.26	1.04	412
S-SN	24.6	0.17	68.0	61.0	2.48	1.11	–
L-SN	52.1	0.16	74.1	78.5	1.51	0.94	–
R-MSM	81.8	0.11	73.0	93.9	1.15	0.78	–
H-MSN	190.7	0.22	179.5	215	1.13	0.83	–

*R*_*e*_ and *σ* represent average particle radius and geometric standard deviation from TEM size distributions log-normal fit. Number of studied particles and adj. *R* ^2^ values can be found from supplementary material. *R*_*g*_ is radius of gyration from SLS and *R*_*h*_ hydrodynamic radius from DLS. SSA_BET_ is specific surface area calculated from nitrogen sorption measurements according to BET theory

### Static Light Scattering

**Fig. 4:**
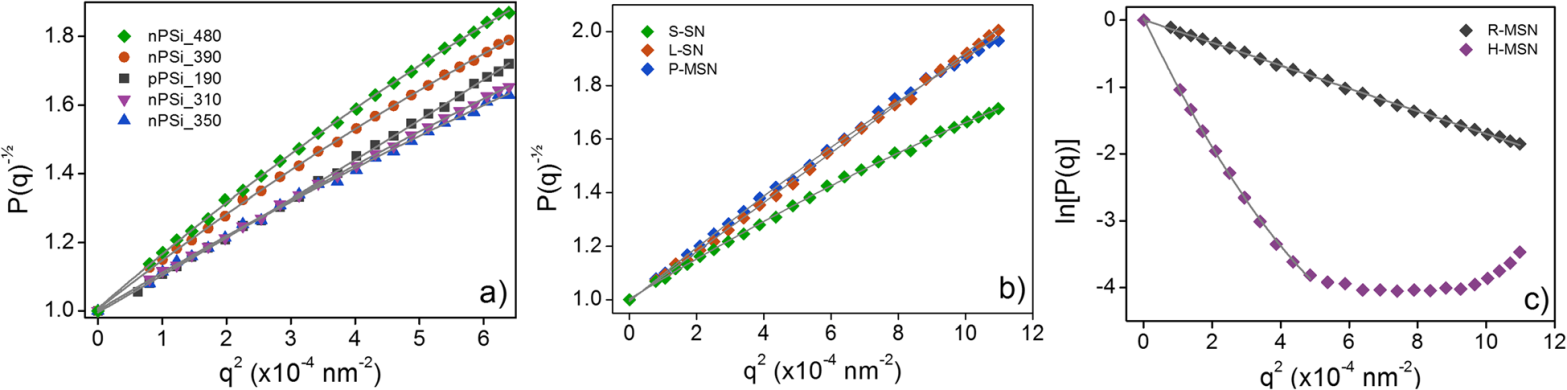
Measured scattering functions *P*(*q*) as a function of squared scattering vector *q* ^2^ and fits for the determination of radius of gyration for the nanoparticles. Debye-Bueche fits were used for PSi nanoparticles (**a**) and for S-SN, L-SN, and P-MSN (**b**). Guinier fits (**c**) were used for the R-MSN and H-MSN

Debye-Bueche fit was used for the SLS result analysis of PSi nanoparticles (Fig. 4). The square root of inverse scattering function (*P*(*θ*))^− ½^ was plotted against the scattering vector *q* ^2^, and second order polynomial fitting was done. In Debye-Bueche plot, the radius of gyration (*R*_*g*_) is calculated from the first order term of the fit. Results can be found from Table 3.

Two different scattering behaviors of silica nanoparticles were observed (Fig. 4). In case of P-MSN, L-SN, and S-SN, the scattering resembled the scattering of PSi nanoparticles and Debye-Bueche fit was used. The scattering of sample R-MSN and H-MSN resembled more a scattering of spherical nanoparticles and Guinier fit was used. In Guinier fit, logarithm of scattering function ln (*P*(*q*)) is plotted against scattering vector *q* ^2^ and the *R*_*g*_ again is calculated from the first order term of the polynomial fit. In the case of H-MSN, Guinier plot was done to *q* ^*2*^–values below 5.10^–4^nm^–2^. Scattering behavior above this resembles the scattering of non-porous hollow silica nanoparticles, and it is typical for core-shell particles [43] but does not fit to Guinier model. All the calculated *R*_*g*_ values for silica nanoparticles are shown in Table 3.

The scattering intensity from fractal particles depends on the scattering angle and fractal dimension (*d*_*f*_) according to power law $P(q)={q}^{-{d}_f}$, when *q* > *R*_*g*_^−1^ [36]. In order to compare fractal dimensions of studied samples, SLS results were fitted to this model (Additional file 1: Figure S6). Fractal dimensions were 1.27–1.48 for PSi nanoparticles. This resembles a scattering of elongated structure or 2D object with fractal surface [36]. In case of silica nanoparticles, the *d*_*f*_ gets values 1.17, 1.53, 1.65, and 2.79 for S-SN, P-MSN, L-SN, and R-MSN, respectively. First three of these are characteristic for fractal agglomerates. The high value in case of R-MSN refers to spherical (perhaps non-fractal) nature of the studied sample.

SLS data is often presented in so-called Kratky plot, where shape and structure of scatterers can be compared to theoretical models presented above. No additional conclusions were made from these plots, but results are shown in Additional file 1: Figure S7.

### Dynamic Light Scattering

**Fig. 5:**
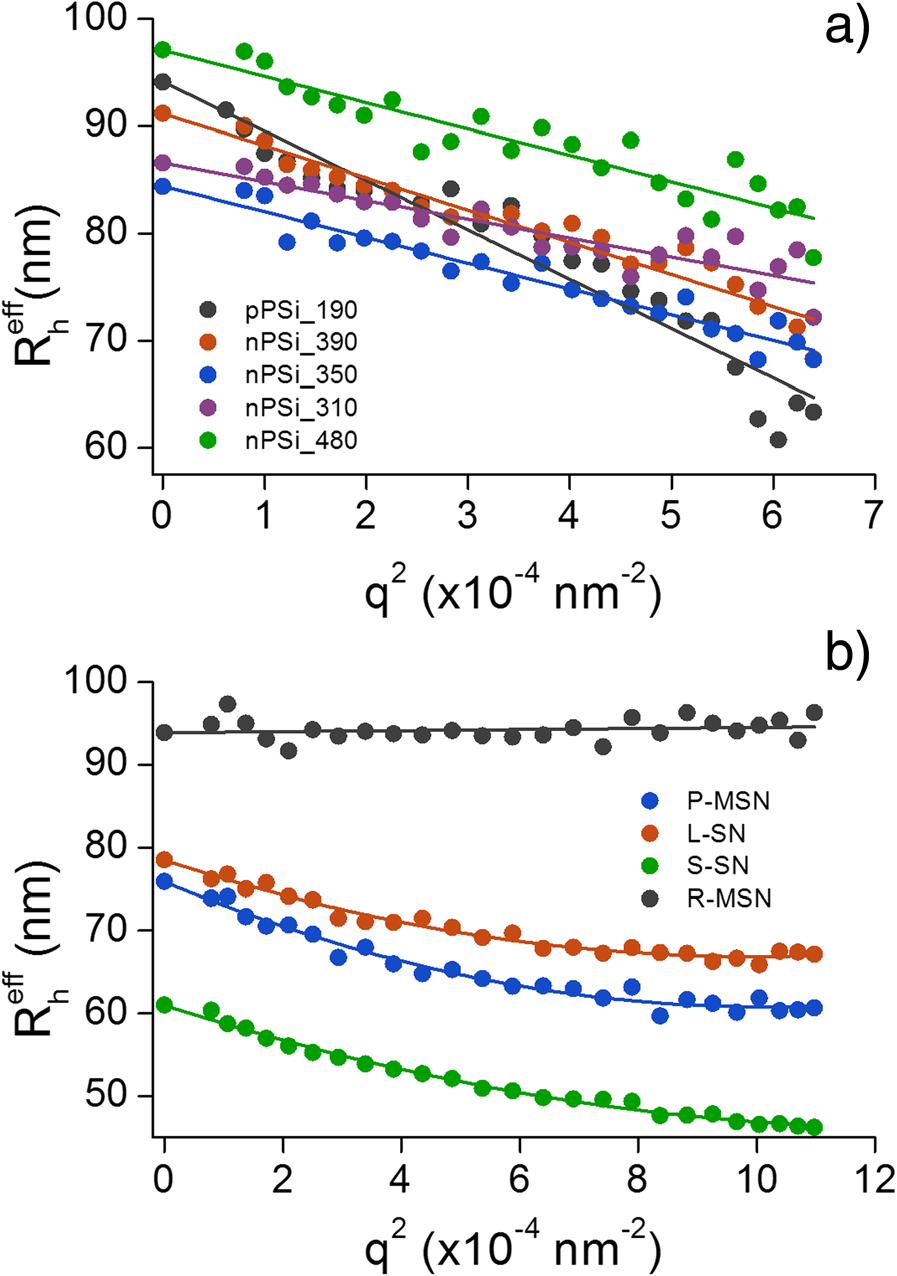
The angular dependence of effective hydrodynamic radius of PSi (**a**) and silica (**b**) nanoparticles. Polynomial or linear fits are made in order to extrapolate the data to *q* = 0

Hydrodynamic sizes of studied nanoparticles were obtained with multiangle DLS measurements. Effective hydrodynamic radius *R*_*h*_^eff^ was plotted against the squared scattering vector *q* ^2^ and the linear or polynomial fit was used in order to extrapolate the data to zero angle (Fig. 5). True hydrodynamic radiuses *R*_*h*_ are tabulated in Table 3.

The decrease of average particle size when the scattering angle or scattering vector is increased was observed for all PSi samples and L-SN, S-SN, and P-MSN samples. This is a well-known phenomenon relating to the polydispersity of suspension and particle size itself [19]. The polydispersity effect arises from the fact that large particles (*R* > *λ*/20) are scattering more light toward the low than high angles. This way, the contribution of big particles at low angles is greater when the average particle size is calculated. In case of small particles (*R* < *λ*/20), the scattering intensity is considered isotropic. If the sample is polydisperse, but all the particles are small, the polydispersity is not affecting angular dependence and *R*_*h*_^eff^. In the case of PSi nanoparticles, we used red laser (637 nm) which means that maximum particle size for isotropic scattering is 32 nm. For silica nanoparticles, blue laser (488 nm) was used so the limit is 25 nm. In one sample (S-SN) only, the primary particle radius was near these limits but the *R*_*h*_ is far above the limit as can be observed from Fig. 5. Owing to these observations, it is considered safe to say that if there is polydispersity in the studied samples, the polydispersity has an effect to the angular dependence of *R*_*h*_^eff^.

Regarding PSi nanoparticles, the differences on the slope of *R*_*h*_^eff^ were observed. Negative slope is the steepest for pPSi_190 particles and lowest for nPSi_310 particles, and it correlates weakly with the width of TEM size distribution *σ* (Additional file 1: Figure S8). This indicates that the major part of the angular dependence of *R*_*h*_^eff^ could be explained with the polydispersity of primary particles.

Silica samples S-SN, L-SN, and P-MSN showed a similar *R*_*h*_^eff^ angular dependence than PSi nanoparticles, but samples R-MSN and H-MSN showed no angular dependence at all. Because of the very different size scale, H-MSN data is not shown. Here, the slopes do not correlate with *σ*, so it cannot be attributed to polydispersity of primary particles (Additional file 1: Figure S9).

### Zeta Potential

Zeta potential distributions of studied nanoparticles in methanol were measured with electrophoretic light scattering (Additional file 1: Figure S10). PSi nanoparticles showed similar zeta potential, ranging from −48 to −57 mV. Silica nanoparticles on the other hand showed large differences on zeta potential, depending on the particle type and fabrication method. S-SN and H-MSN had a negative zeta potential of approximately −46 mV. L-SN, P-MSN, and R-MSN had positive zeta potential of 41, 67, and 75 mV, respectively.

## Discussion

### Average Radiuses and Overestimation Hydrodynamic Radius

All measured radiuses are tabulated on Table 3 with overestimations between hydrodynamic radius and TEM primary particle radius (*R*_*h*_/*R*_*e*_). This overestimation was significant for almost all PSi samples, and variation between samples was relatively low (overestimation ranged from 31 to 65%). In case of silica nanoparticles, the variation of the overestimation between particle types was considerable as the smallest overestimation (13%) was measured for the sample H-MSN and highest (148%) for the sample S-SN.

### Effect of Polydispersity

When comparisons between DLS and TEM radiuses are made, it must be noted that these techniques and particle size distributions are fundamentally different. TEM distribution datapoint is based on the number of the particles in a studied size class whereas DLS distribution is based on the intensity of light that is scattered by the studied size class. Some overestimation is therefore expected. For a polydisperse sample, this might be significant, and in case of PSi nanoparticles, which all have a wide size distribution, it is possible that the overestimation is explained by this difference between techniques. But in this case, we should observe correlation between the overestimation and the polydispersity.

If we take the geometric standard deviation (*σ*) of the TEM size distributions to describe the polydispersity of primary particles, we can clearly see that these are not correlating with overestimations (Additional file 1: Figure S8). This is the case also with the silica particles (Additional file 1: Figure S9). On the other hand, if we take the slope of *R*_*h*_^eff^ from Fig. 5 to describe the polydispersity of the sample in its native colloidal state and compare that with overestimations, there is no correlation among PSi nanoparticles (Additional file 1: Figure S8), but a weak trend can be seen among silica nanoparticles (Additional file 1: Figure S9). Since the standard deviation describes the polydispersity of primary particles but DLS measurements takes also agglomerates into account, in case of silica nanoparticles, the overestimation might be caused by the polydispersity arising from the agglomeration. Regarding PSi nanoparticles, the overestimation cannot be explained by the difference between number and intensity weighted particle size distribution alone.

### Nanoparticles’ Porosity and Stability

As explained in experimental part, *R*_*h*_ is calculated from the diffusion of the particles in liquid environment. Owing to the fact that this size actually describes the dimension of the sphere having a same diffusion coefficient *D*_*t*_ than the studied particle, the *R*_*h*_ is also called equivalent radius. One typical phenomenon, which is slowing the particle diffusion in the solution and this way leads to increased *R*_*h*_, is the solvation layer around the particle. This effect is not very significant with large nanoparticles since the solvation layer is typically only a few nanometers. More important phenomenon is the structure of the particle. This is previously studied more with different kind of fractal aggregates of very small silica nanoparticles where the fractal size, fractal dimensions, and the specific surface area are playing a role [44].

#### Silica Nanoparticles

**Fig. 6:**
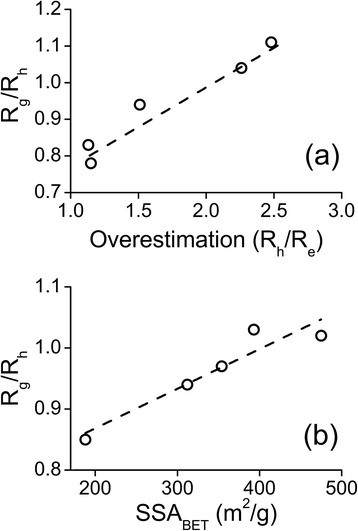
R_g_/R_h_ as a function of R_h_/R_e_ overestimation for silica nanoparticles (**a**). R_g_/R_h_ as a function of specific surface area of PSi nanoparticles (**b**). *Dashed lines* represent a linear fit to the data

The easiest way to analyze the morphology of the nanoparticles in LS experiments is to look into the ratio of radius of gyration and hydrodynamic radius *R*_*g*_/*R*_*h*_ (Table 3). Investigation of this value on respect of the size overestimation reveals interesting behavior of silica nanoparticles and the correlation is clear (Fig. 6 and Additional file 1: Figure S9). The bigger the overestimation is, the larger is also the *R*_*g*_/*R*_*h*_. Noteworthy is also the result that the *R*_*g*_/*R*_*h*_ value does not correlate with the porosity of silica nanoparticles. *R*_*g*_/*R*_*h*_ is 1.11 and 0.94 for non-porous S-SN and L-SN and 0.78 for porous R-MSN. Since high *R*_*g*_/*R*_*h*_ value can be connected to more complex fractal nature [44], this further confirms the connection between overestimation and agglomeration. In addition, the DLS slope, which can be connected to the polydispersity arising from the agglomeration, correlates with *R*_*g*_/*R*_*h*_ value (Additional file 1: Figure S9).

This is a significant observation, since the common assumption is that hydrodynamic radius and polydispersity index (PdI), obtained from the one angle DLS measurement with Cumulants analysis, could reveal if the agglomerates are present. This is the deduction that we have also made during the preparation of nanoparticle samples.

Zeta potential is an important parameter explaining the stability of colloid against the agglomeration. We measured zeta potential in methanol in order to compare it to LS results (Additional file 1: Figure S10). It seems that zeta potential cannot explain the agglomeration of silica nanoparticles as one of the agglomerated sample (P-MSN) has also one the highest zeta potential. Besides zeta potential, the stability of non-aqueous colloid depends also on particle size [45]. Since all particles have sufficiently high zeta potential, the particle size plays a considerable role on stability which can also be seen in the results since the largest silica samples R-MSN and H-MSN seem to be the most stable ones.

#### PSi Nanoparticles

Overestimation of hydrodynamic radius, when compared to TEM radius, was less pronounced in the case of PSi nanoparticles, but still clear. The primary particle size *R*_*e*_ varied less between samples, and the surface chemistry was the same in all the samples, which is shown also in similar zeta potentials (Additional file 1: Figure S10). A very weak correlation between overestimation and *R*_*g*_/*R*_*h*_ might also be seen (Additional file 1: Figure S8) with PSi nanoparticles, but stronger explanation seems to be the primary particle size. These are indicating agglomeration, which was the case with silica nanoparticles, but no similar correlation between *R*_*g*_/*R*_*h*_ and DLS slope was found. On the contrary, as explained before, standard deviation correlates with the DLS slope, which emphasizes the polydispersity of primary particles, not agglomerates. Because of these conflicting or too weak correlations, no conclusive explanation for the overestimation was found.

Nevertheless, a strong correlation between *R*_*g*_/*R*_*h*_ and specific surface area, SSA_BET_ (Fig. 6) was found for PSi nanoparticles. The complex nature of PSi nanoparticles is evident from TEM pictures, but the effect of structure to LS results has not been noticed before. This is a very intriquing result since it might give a way to characterize the structure of PSi nanoparticles in solution. Strong impact of specific surface area to morphology sensitive *R*_*g*_/*R*_*h*_, and weak correlation between overestimation and *R*_*g*_/*R*_*h*_ gives reason to speculate that specific surface area might explain the overestimation, but there are probably other factors also contributing which were not under the scope of this study.

## Conclusions

The size of silicon-based mesoporous nanoparticles was measured with three different techniques: transmission electron microscope (TEM), dynamic light scattering (DLS), and static light scattering (SLS). Primary particle radius *R*_*e*_ was measured with TEM, hydrodynamic radius *R*_*h*_ with DLS, and radius of gyration *R*_*g*_ with SLS. Recently, DLS has become the most common method to characterize nanoparticle sizes and big differences between DLS and TEM sizes have been observed. These differences are normally attributed to the fundamental difference between intensity and number weighted particle size distributions and the differences between dry and hydrodynamic radius of particles. In this report, we studied the sizes of porous silicon (PSi) nanoparticles and mesoporous silica nanoparticles (MSN). As expected, we also observed remarkable difference between hydrodynamic radius and primary particle radius. Depending on the primary particle size, zeta potential, and porosity, the overestimation varied from 13 to 148%.

The overestimation of the silica nanoparticles’ *R*_*h*_ was attributed to agglomeration of primary particles. This was the case despite the careful selection of the dispersion medium. If the porosity plays a role on the measurement, we were not able to distinguish it because of the strong agglomeration effect. The overestimation affected *R*_*g*_/*R*_*h*_ value so that the high overestimation between DLS and TEM yielded also high *R*_*g*_/*R*_*h*_. This could be caused by the fractal nature of the agglomerates.

In case of PSi nanoparticles, the clear correlation between *R*_*g*_/*R*_*h*_ and the specific surface area was observed. Overestimation of *R*_*h*_ was also observed, and the variation of the overestimation between PSi samples was smaller. The evidences are pointing to the direction that we were able to measure primary particles with both LS techniques, but no single explaining factor for the overestimation could not be found.

The measurement of the multiangle LS was found to be useful for characterization of mesoporous nanoparticles and could be used to gain information on particle size, agglomeration, and possibly porosity also. It is evident that there are drawbacks also on SLS and DLS measurements, but the careful measurement of angular dependence of the *R*_*h*_ and the comparison of the result to radius of gyration *R*_*g*_ can be used to obtain more detailed information of the particle size distribution and morphology of the studied sample in colloidal state.

## Additional file

Additional file 1: Contains following supplementary materials: fabrication of porous silicon nanoparticles, fabrication of silica nanoparticles, summary of silica nanoparticles' preparation conditions, summary of log-normal fitting results, absorbance of used nanoparticles, nitrogen sorption isotherms, additional TEM graphs from silica nanoparticles, fractal dimension analysis for SLS results and Kratky plots, all the studied correlations and measured zeta potential distributions. (DOCX 11779 kb)

## Abbreviations

DLS: Dynamic light scattering
LS: Light scattering
L-SN: Large silica nanoparticle
MSN: Mesoporous silica nanoparticle
P-MSN: Mesoporous silica nanoparticles with pores parallel to each other
PSi: Porous silicon
R-MSN: Radial pore mesoporous silica nanoparticle
SLS: Static light scattering
SN: Silica nanoparticle
S-SN: Small silica nanoparticle
TEM: Transmission electron microscope
