# Supplementary material for: Size, Stability, and Porosity of Mesoporous Nanoparticles Characterized with Light Scattering
Source: Nanoscale Res Lett. 2017 Jan 25;12:74. doi: 10.1186/s11671-017-1853-y (PMC5267583; doi:10.1186/s11671-017-1853-y)
Supplement: Supplementary file 1 — Contains following supplementary materials: fabrication of porous silicon nanoparticles, fabrication of silica nanoparticles, summary of silica nanoparticles' preparation conditions, summary of log-normal fitting results, absorbance of used nanoparticles, nitrogen sorption isotherms, additional TEM graphs from silica nanoparticles, fractal dimension analysis for SLS results and Kratky plots, all the studied correlations and measured zeta potential distributions. (DOCX 11779 kb) [file 11671_2017_1853_MOESM1_ESM.docx]

**Additional file**

Size, stability and porosity of mesoporous nanoparticles characterized with light scattering

Martti Kaasalainen^a^*, Vladimir Aseyev^b^, Eva von Haartman^c^, Didem Sen Karaman^c^, Ermei Mäkilä^a^, Heikki Tenhu^b^, Jessica Rosenholm^c^ and Jarno Salonen^a^

*^a^ Laboratory of Industrial Physics, Department of Physics and Astronomy, University of Turku, FI-20500 Turku, Finland, martti.kaasalainen@utu.fi, emmaki@utu.fi, jarno.salonen@utu.fi*

*^b^ Laboratory of Polymer Chemistry, Department of Chemistry, University of Helsinki, FI-00014 HY Helsinki, Finland, vladimir.aseyev@helsinki.fi, heikki.tenhu@helsinki.fi,*

*^c^ Pharmaceutical Sciences Laboratory, Faculty of Science and Engineering, Åbo Akademi University, FI-20520 Turku, Finland, ehaartma@abo.fi, dsen@abo.fi, jerosenh@abo.fi*

** Corresponding author: tel. +358 (0)2 333 5736*

1. Fabrication of porous silicon nanoparticles

Porous silicon nanoparticles were fabricated with electrochemical anodization. Mixture (1:1) of hydrofluoric acid (40 %, Merck KGaA, Germany) and ethanol ($\geq$99.5 vol-%, Altia Oyj, Finland) was used as an electrolyte. Monocrystalline Si(100) wafer is used as an anode of the electrochemical cell and etching takes place on the silicon surface when current is applied.

In order to fabricate porous silicon nanoparticles with different porosities, two different techniques were used. In the first method, lower current density pulses are used for the formation of mesoporous layer and higher current density pulses are used for creating fracture layers between the mesoporous layers. Target of this approach is to control the size and yield of nanoparticles. Multilayered structure was etched on conductive p^+^-type silicon wafer in order to achieve PSi nanoparticles with a lower porosity and bigger pore size. In second method constant current was used to etch n^+^-type silicon wafer. In this case, high intensity illumination was used in order to create charge carriers, which are essential for etching process, to the wafer. Lightning conditions and currents were altered so that different kind of porosities were obtained.

After etching, silicon surface was stabilized with thermal oxidation at 300°C air for a 2 h. Size reduction was made, in case of less porous p^+^-type films, by wet milling the PSi films in ethanol with a planetary ball mill. Porous n^+^-type silicon was etched on silicon wafer without lift‑off pulse and the size reduction was made mechanically (Mirka EcoWet P2500 with silicon carbide microparticles). Water was used as a grinding medium.

After grinding and milling, formed particles are very polydisperse. Medium was changed into ethanol and samples were centrifuged in order to separate nanoparticles. Depending on the particle batch, the acceleration from 1600 g to 4000 g was used. Medium change into methanol was made by centrifuging nanoparticles with high acceleration (17 000 g) and replacing the supernatant. Replacement was made two or three times, depending on the original sample size.

1. Fabrication of silica nanoparticles

Non-porous silica nanoparticles (SN) were synthesized through sol-gel silica polymerization. Two different particle batches with different sizes and surface charge were prepared varying the amount of aminosilane used in the synthesis. The smaller nanoparticles are abbreviated S-SN and the larger L-SN. In a typical synthesis procedure 100 ml absolute ethanol (EtOH) and 6 ml ammonia (33 wt%) were mixed in a beaker. 3.4 ml tetraethoxyorthoslicate (TEOS), 250 µl fluorescein isothiocyanate (FITC) (1 mg/ml in DMF) and 35 µl (S-SN) or 350 µl (L-SN) 3-aminopropyltriethoxysilane (APTS) were mixed in a separate beaker and added to the reaction solution under vigorous stirring. The mixture was stirred in room temperature (RT) over night. The particles were subsequently separated by centrifugation. After addition of a few drops of 1 M HCl, which was added to the reaction mixture in order to destabilize it. The particles were further washed on an ultrasonication bath first one time with deionized water (MilliQ), then two times with acetone. The particles were finally separated and dried in vacuum at 30°C.

Mesoporous silica nanoparticles with radial pores (R-MSN) were prepared in the following way. 600 mg CTAB, 155 ml deionized water (MilliQ), 58 ml absolute ethanol and 800 µl ammonia (33 wt%) was mixed in a beaker at RT. 1.2 ml TEOS and 125.6 µl (10 mol-%) APTES was mixed in a separate beaker and added to the mixture under vigorous stirring. The resulting mixture was stirred over night at RT. The particles were separated by centrifugation and extracted three times with an ammonium nitrate solution (2 g/100 ml EtOH) for 30 min. Finally, the particles were washed with acetone, separated by centrifugation and immediately redispersed and stored in acetone.

Mesoporous silica nanoparticles with parallel pores (P-MSN) were prepared in the following way. 4.68 mg cetyltrimethylammoniun bromide (CTAB), 720 ml deionized water (MilliQ), 120 ml ethylene glycol (EG) and 22 ml ammonia (33 wt%) were mixed in a glass beaker for 30 min. at 50°C. 5.7 ml TEOS and 1.05 ml APTS were mixed in a separate beaker and added under vigorous stirring. The reaction mixture was stirred at 50°C for 2 h and then aged at static conditions at 50°C over night. The structure-directing agent was subsequently extracted (3x30 min) with acidic ethanol (360 ml EtOH; 45 ml 38% HCl) and particles separated by centrifugation. The particles were finally redispersed and stored in anhydrous acetone.

**Table S1** Summary of silica nanoparticles’ preparation conditions. Detailed description of the synthesis can be found from supplementary material (S2) and from ref. [1].

| **Sample** | **Base solution** | **Additives** | | **Temp.** | **Mixing** | **Extraction** | **Storing** |
| --- | --- | --- | --- | --- | --- | --- | --- |
| S-SN | EtOH/ammonia | TEOS+FITC | 1 mol% APTES | RT | Stirring, over night | - | Dried |
| L-SN | EtOH/ammonia | TEOS+FITC | 10 mol% APTES | RT | Stirring, over night | - | Dried |
| R-MSN | CTAB/di‑water/  EtOH/ammonia | TEOS | 10 mol% APTES | RT | Stirring, over night | 3x30 min @  (NH4)(NO3) - EtOH | Acetone |
| P-MSN | CTAB/di‑water/ethylene glygol/ammonia | TEOS | 20 mol% APTES | 50°C | Stirring 2h + static over night | 3x30 min @  HCl - EtOH | Acetone |
| H-MSN | CTAB/di‑water/  NaOH/ethanol | TEOS | 10 mol% APTES / 1.2 mol % Decane /  1.8 mol % (1, 3, 5) Trimethylbenzene | RT | Stirring, over night + hydrothermally treated at 70^◦^C for 48h. | Calcination at 550^◦^C for 2.5 h. | Dried |
| [1] D. Sen Karaman, T. Gulin-Sarfraz, J. Zhang, J.M. Rosenholm, Mater. Lett. 143 (2015) 140–143. | | | | | | | |

**Table S2** Summary of log-normal fitting results with Origin 8^a^

| **Sample** | **R_e_** | σ | **N** | **Adj. R^2^** |
| --- | --- | --- | --- | --- |
| nPSi_390 | 55.3 | 0.35 | 345 | 0.995 |
| nPSi_350 | 57.5 | 0.30 | 344 | 0.997 |
| nPSi_480 | 69.3 | 0.29 | 75 | 0.988 |
| pPSi_190 | 72.1 | 0.36 | 350 | 0.990 |
| P-MSN | 33.6 | 0.19 | 1010 | 0.999 |
| S-SN | 24.6 | 0.17 | 456 | 1.000 |
| L-SN | 52.1 | 0.16 | 432 | 1.000 |
| R-MSN | 81.8 | 0.11 | 681 | 1.000 |
| H-MSN | 190.7 | 0.22 | 612 | 0.998 |
| ^a^ $R_{e}$, σ and $Adj. R^{2}$ represent average particle radius, geometric standard deviation and adjusted $R^{2}$ value of the log-normal fit. N is the amount of particles measured for the size determination. | | | | |


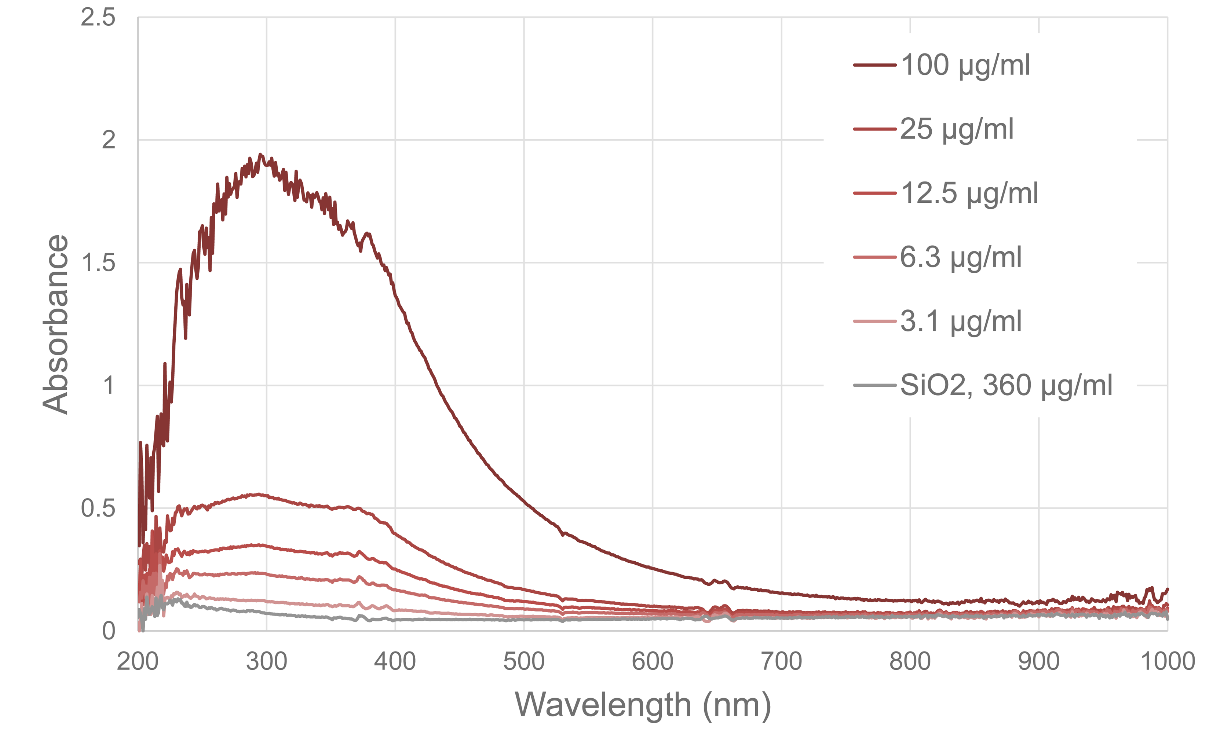


Figure S1. Absorbance of used nanoparticles, PSi (red) and MSN (grey), in methanol with different concentrations. Measurements were made in well plate with UV-vis spectrophotometer (Labrox, Finland).


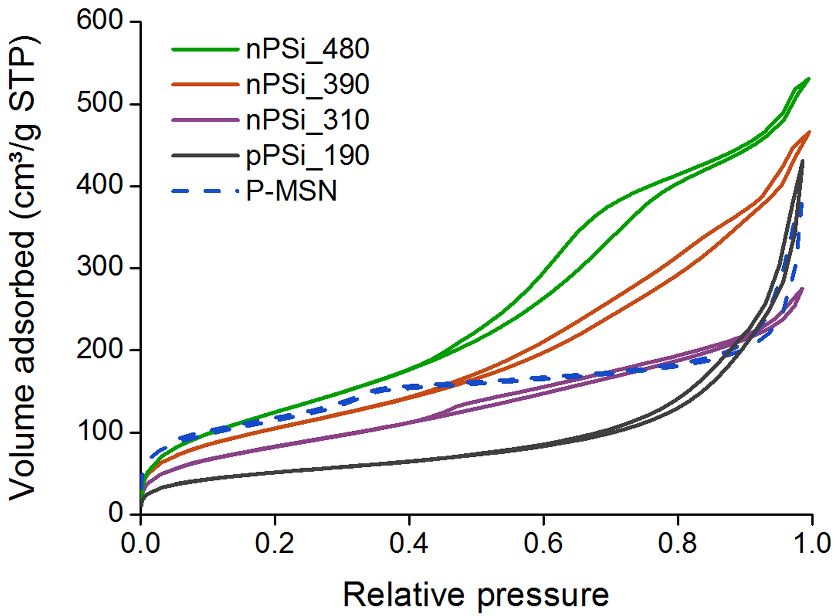


Figure S2. Nitrogen sorption isotherms for selected nanoparticles.

| 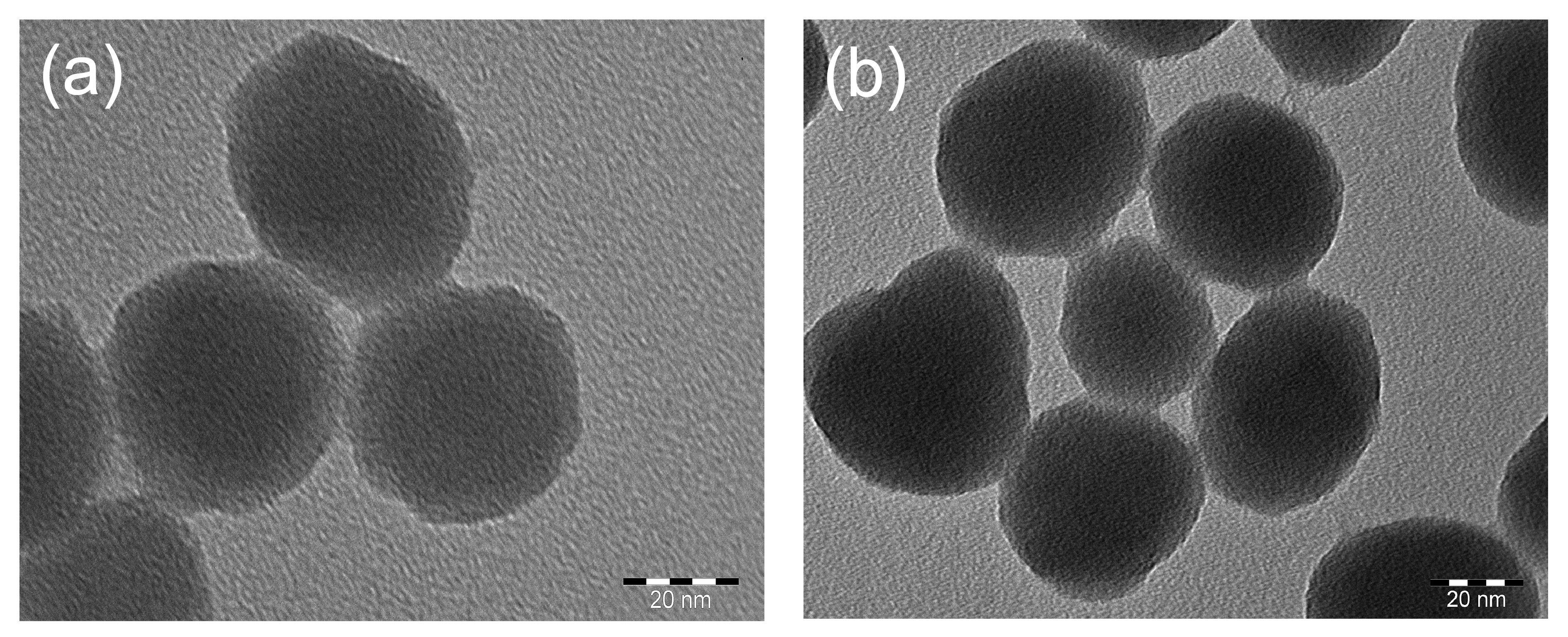 |
| --- |
| Figure S3. Small non-porous silica particles (S-SN) |
| 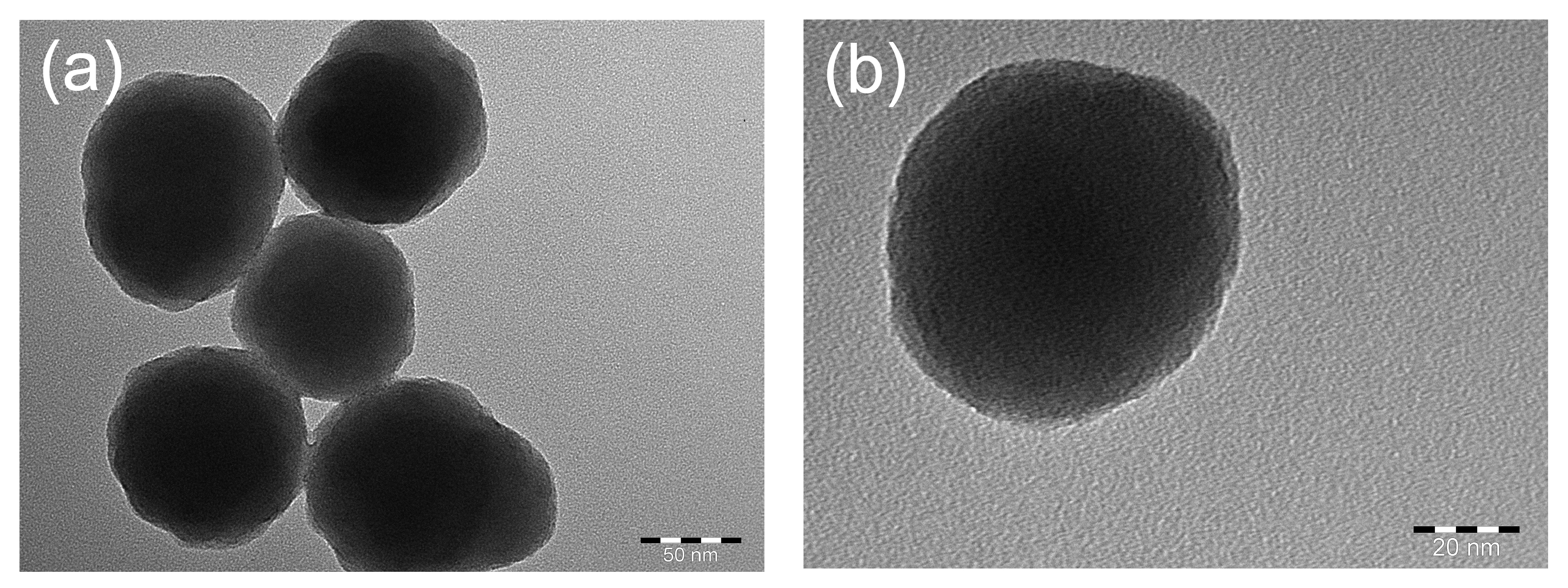 |
| Figure S4. Large non-porous silica particles (L-SN) |
| 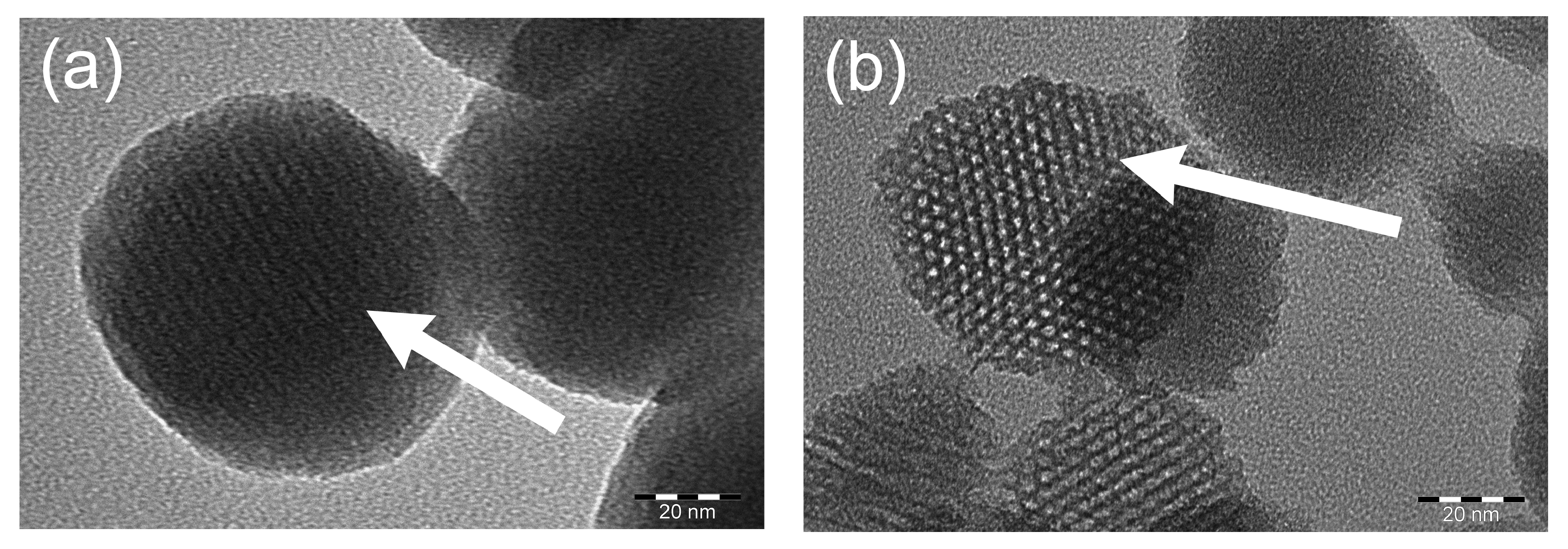 |
| Figure S5. Mesoporous silica particles where pores are parallel to each other (P-MSN). TEM image reveals the pore structure when the pores are perpendicular to electron beam (a) or parallel to electron beam (b). |

| 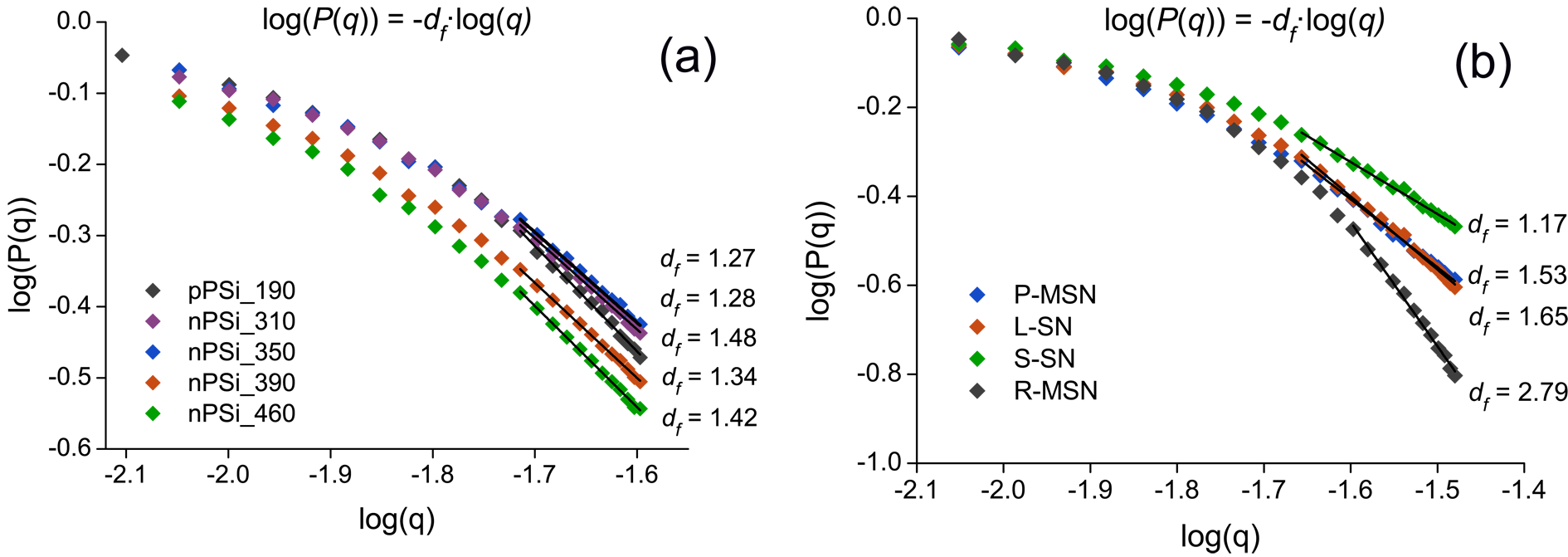 |
| --- |
| Figure S6. Fractal dimension analysis from SLS data. PSi nanoparticles on figure (a) and silica nanoparticles on figure (b). |

| 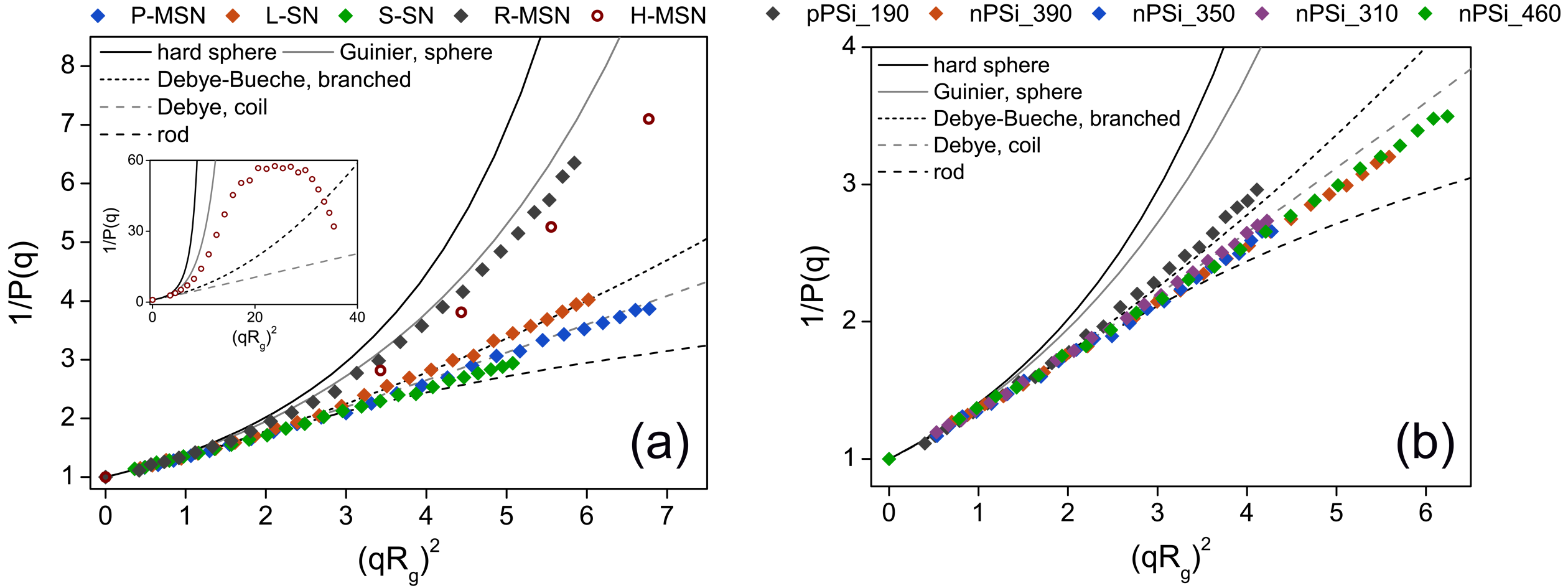 |
| --- |
| Figure S7. SLS-results presented in a Kratky plots. Solid lines represent a theoretical scattering functions (they are not fits) and dots are data points from measurements. Radially porous R-MSN (a) resembles the scattering behavior of a sphere, whereas P-MSN, L-SN and S-SN particles fit more to Debye-Büeche model. Inverse scattering function of H-MSN (inset (a)) shows a maximum, which was attributed to scattering of hollow sphere. All PSi nanoparticles (b) are very close to a Debye-Büeche scattering model. |


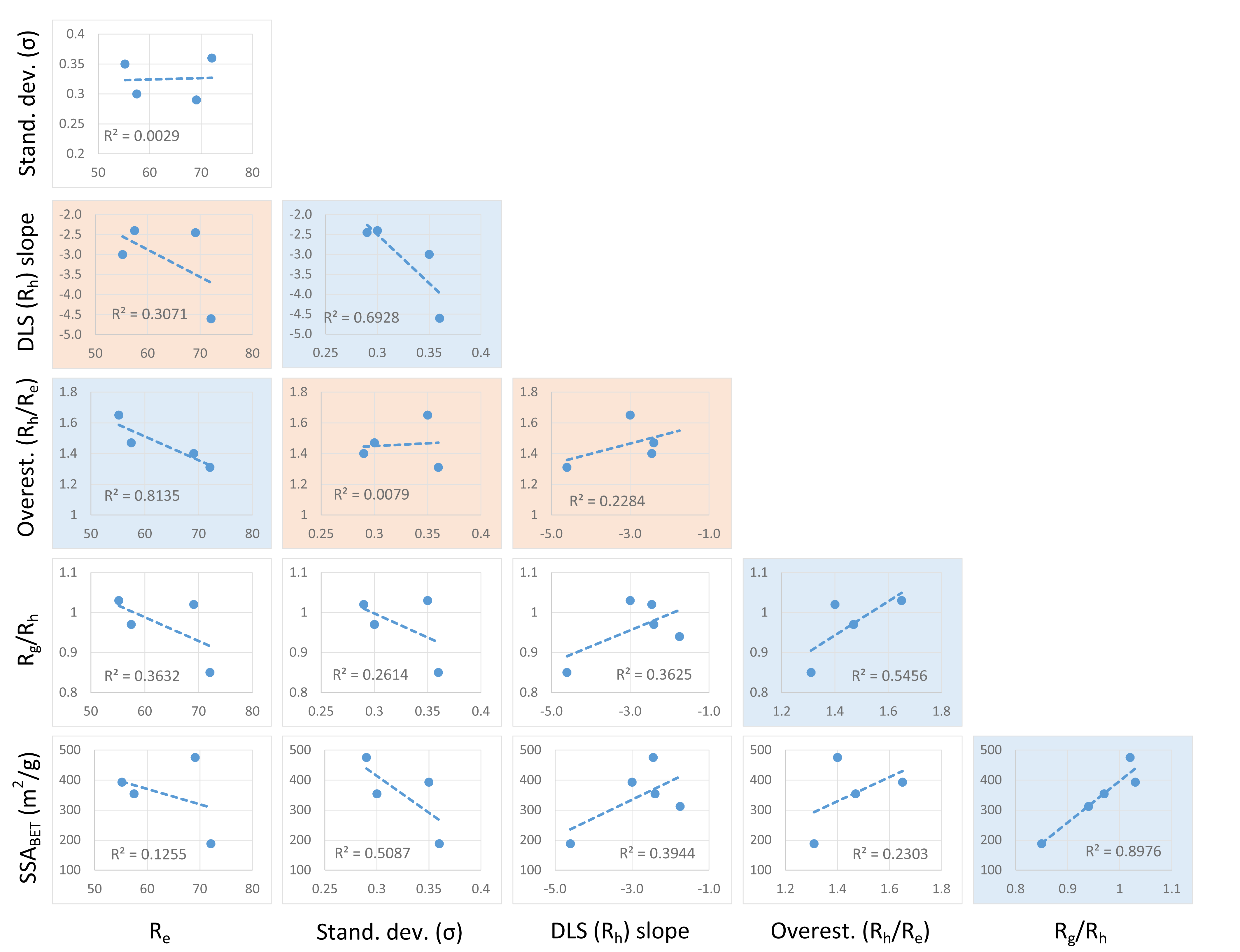


Figure S8. Correlation of the studied parameters in the case of PSi nanoparticles.


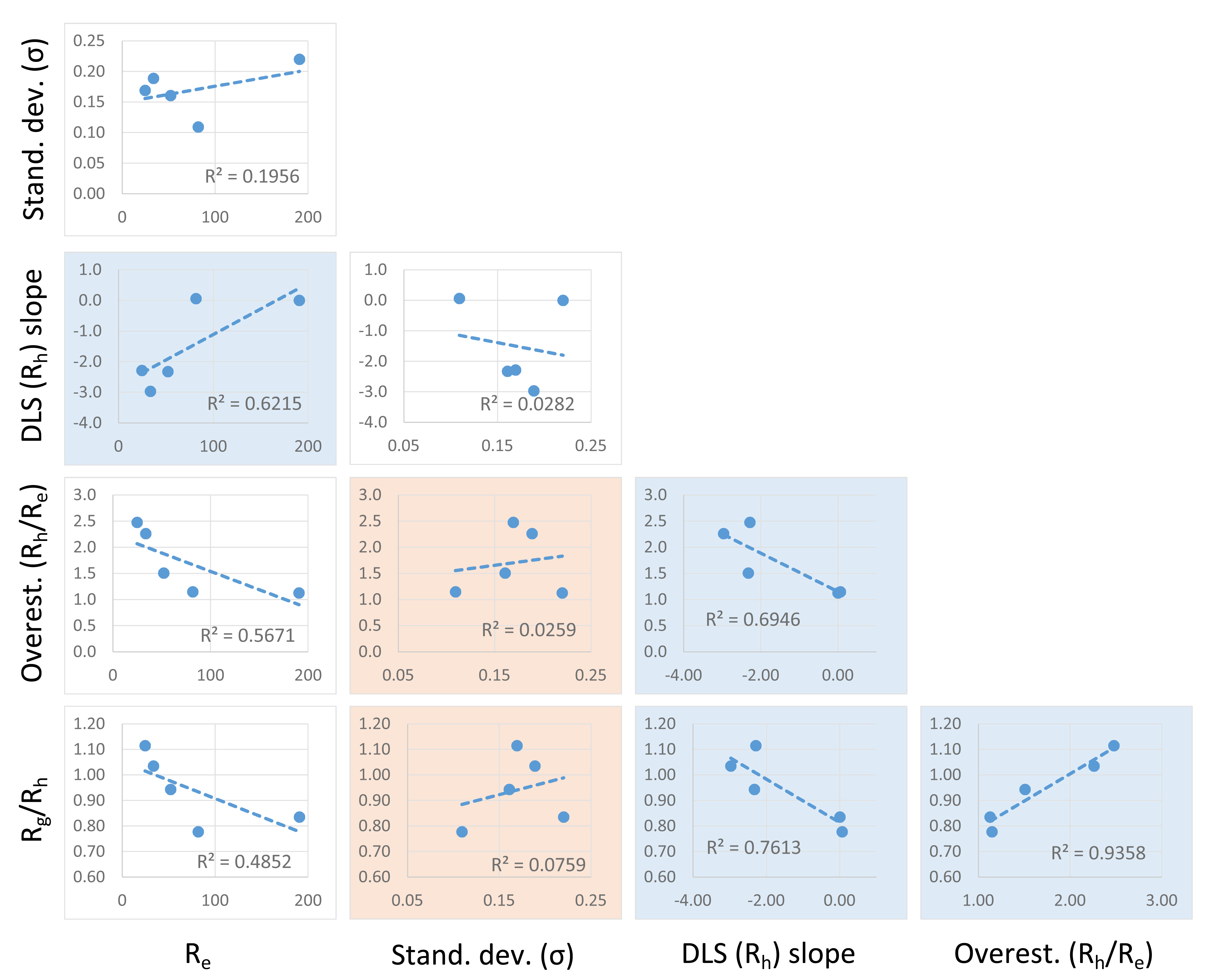


Figure S9. Correlation of the studied parameters in the case of silica nanoparticles.


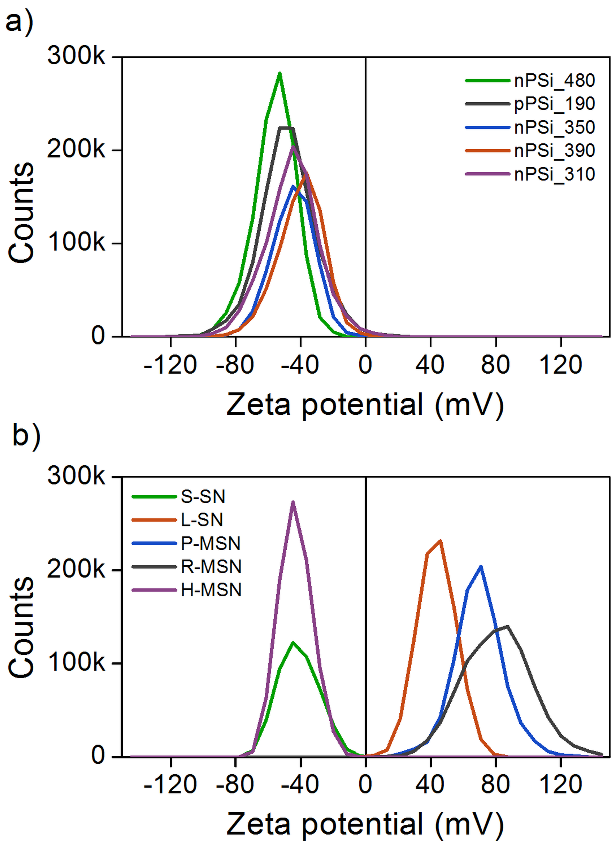


Figure S10. Measured zeta potential distributions of PSi (a) and silica (b) nanoparticles.
